# Effectiveness of Technological Interventions for Older Adults With Parkinson Disease: Systematic Review

**DOI:** 10.2196/53431

**Published:** 2024-09-09

**Authors:** Roberta Bevilacqua, Marco Benadduci, Federico Barbarossa, Giulio Amabili, Valentina Di Donna, Clotilda Martella, Giuseppe Pelliccioni, Giovanni Renato Riccardi, Elvira Maranesi

**Affiliations:** 1 IRCCS INRCA Ancona Italy

**Keywords:** technological intervention, Parkinson disease, randomized controlled trail, older adults, efficacy

## Abstract

**Background:**

Among the older population, Parkinson disease (PD) stands out as a leading contributor to disability. Clinically, the foremost objectives in managing PD involve proactively delaying and preventing disability. Understanding the pivotal role of gait and balance in daily functionality holds substantial clinical significance, signaling imminent disability and prompting a reevaluation of management approaches. A key priority lies in identifying novel and effective interventions for symptoms that substantially contribute to disability.

**Objective:**

This paper presents a systematic review that critically examines the existing body of literature on the use of technology in the rehabilitation of older patients with PD. By synthesizing current evidence, we aim to provide insights into the state of the field, identify gaps in knowledge, and offer recommendations for future research and clinical practice.

**Methods:**

A systematic review of the literature was conducted in September 2023 analyzing manuscripts and papers of the last 5 years from the PubMed, Scopus, Embase, Web of Science, and CINAHL databases following PRISMA (Preferred Reporting Items for Systematic Reviews and Meta-Analyses) guidelines. A total of 14 papers were included. The inclusion criteria are as follows: (1) randomized controlled trial, (2) PD in people aged 65 years and older, and (3) use of technology in the rehabilitation training in the older population.

**Results:**

A large portion of effective interventions relies on the incorporation of technology, particularly through virtual reality exergames. This technology appears to have effects not only on the cognitive aspect but also on the physical domain. The analysis of the results clearly indicates that, in terms of gait and balance performance, the technological intervention outperforms the traditional approach, irrespective of the specific technology employed.

**Conclusions:**

This systematic review seeks to shed light on the evolving landscape of technology-assisted rehabilitation for older individuals with PD. As we delve into the available evidence, we will assess the extent to which technology can serve as a valuable adjunct to conventional therapy, offering new avenues for optimized care and improved outcomes in this growing patient demographic. As we sift through the existing evidence, our goal is to evaluate the potential of technology as a valuable supplement to traditional therapy, presenting fresh opportunities for enhanced care and better outcomes in this expanding patient demographic.

## Introduction

Neurological disorders stand as the primary cause of global disability [1]. Within this category, Parkinson disease (PD) is exhibiting the most rapid increase in disability, fatalities, and prevalence, with an anticipated doubling within the next 2 decades [2]. In Europe, the current PD population exceeds 1.2 million, contributing to an annual economic burden of €13.9 billion (US $15.5 billion) [3]. The annual cost per individual rises with the severity of the condition, while nonmotor symptoms significantly contribute to hospitalizations and institutionalizations. Beyond the challenging aspects of PD itself, individuals with PD grapple with notable comorbidities and a high frequency of falls [4]. Aging is correlated with an accelerated motor progression of the disease; reduced responsiveness to levodopa; heightened severity in gait, posture, and cognitive impairment; and an increased likelihood of developing dementia [5].

With the aging of the global population, the prevalence of PD is on the rise, making it an increasingly significant public health concern. Effective management and rehabilitation strategies are essential to mitigate the debilitating effects of this condition and to enhance the functional independence of older individuals living with PD.

From a clinical perspective, the top priorities in PD management include the delay and prevention of disability [6]. Despite symptomatic relief through medical, surgical, and rehabilitative interventions, older individuals with PD experience a persistent deterioration in disability, marked by a decline in quality of life, diminished functional mobility, decreased performance in daily activities, and worsening neurological impairments. Guidelines suggest early initiation of physical therapy at the onset of the disease for addressing functional decline [7]. However, the evidence regarding its effectiveness in delaying symptom onset or reducing severity is not robust. Recognizing the crucial role of gait and balance in daily function has significant clinical implications, indicating impending disability and necessitating a reassessment of management strategies. The identification of new and effective interventions for symptoms contributing to significant disability is a key priority [8]. In particular, there is a growing body of evidence indicating that exercise and physical activity interventions can slow the rate of functional mobility decline, ultimately improving quality of life, as tertiary prevention solutions [8].

Over the years, advances in technology have revolutionized the field of health care, offering innovative tools and interventions for the diagnosis, monitoring, and treatment of various medical conditions. In recent decades, there has been a growing interest in harnessing the potential of technology to aid in the rehabilitation of patients with PD, particularly in the older population. This shift reflects a broader trend toward incorporating digital solutions into health care, driven by the desire to optimize therapeutic outcomes and improve the overall well-being of patients.

For this reason, recent research [9-11] indicates that balance training delivered through technology leads to performance enhancements that align with noticeable neurobiological changes in the cerebral cortex [9,10]. This underscores the encouraging potential of technological interventions in aiding individuals with PD in managing balance and other motor disorders.

This paper presents a systematic review that critically examines the existing body of literature on the use of technology in the rehabilitation of older patients with PD. By synthesizing current evidence, we aim to provide insights into the state of the field, identify gaps in knowledge, and offer recommendations for future research and clinical practice.

In this comprehensive review, we will explore a wide range of technological applications, including wearable devices, telerehabilitation platforms, virtual reality systems, and robotics, among others. We will evaluate the effectiveness, usability, and safety of these technologies in improving motor function, balance, mobility, and overall quality of life in older patients with PD. Furthermore, we will consider the potential challenges and ethical implications associated with the integration of technology into rehabilitation protocols for this vulnerable population.

## Methods

### Overview

The methodology of this systematic review was based on the PRISMA (Preferred Reporting Items for Systematic Reviews and Meta-Analyses) guidelines (Multimedia Appendix 1 [12]) [13], with the main aim of analyzing the use of technology in the rehabilitation of older patients with PD.

We used the PICO (Population, Intervention, Comparator, Outcome) framework as follows:

- P: Older patients with PD
- I: Rehabilitation program with technology
- C: Control group that receives a traditional rehabilitation program
- O: The efficacy of the treatment

**Figure 1:**
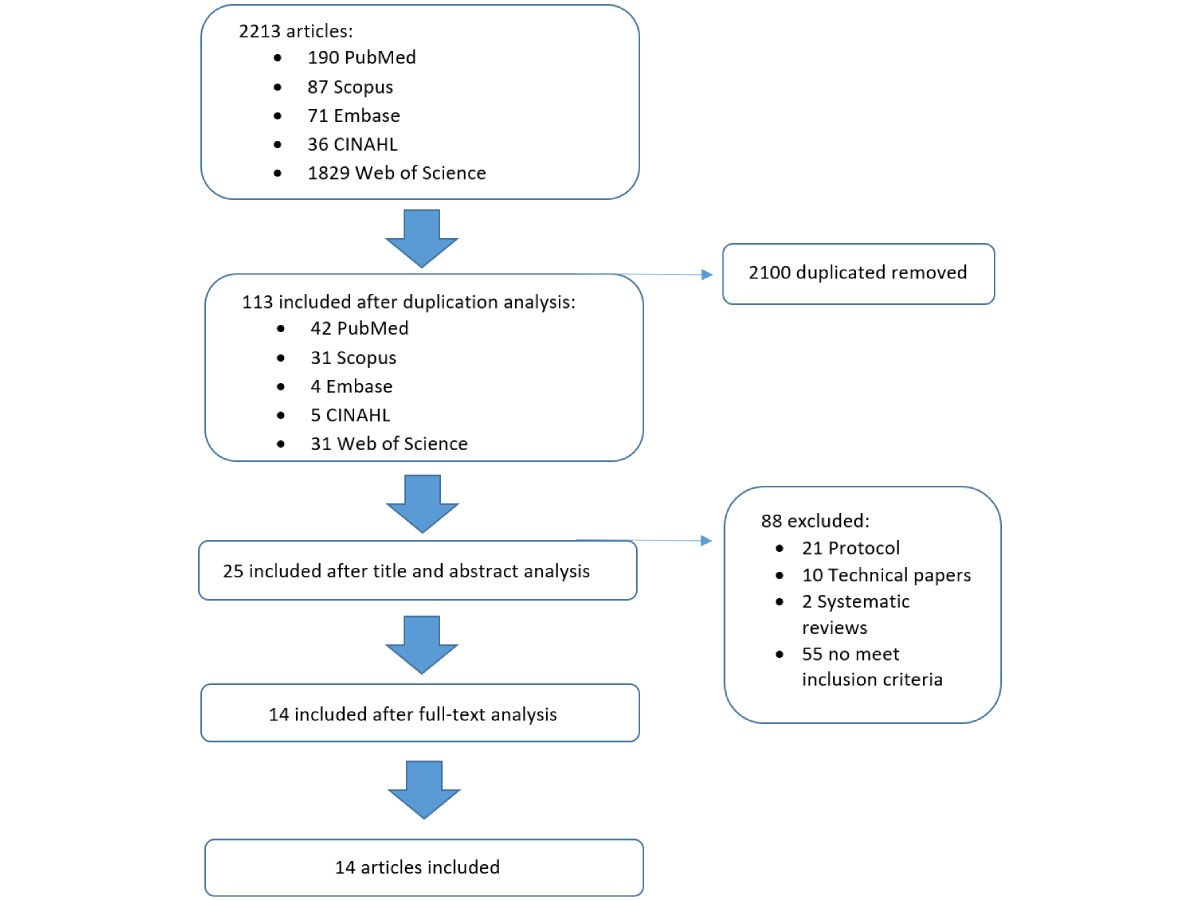
Descriptive analysis of the included clinical studies.

A systematic review of the literature was conducted in September 2023. The data were collected from PubMed, Embase, Scopus, CINAHL, and Web of Sciences, analyzing manuscripts and papers of the last 5 years (from September 2018 to September 2023) in order to obtain the latest evidence in the field. The inclusion criteria were as follows: (1) randomized controlled trial, (2) PD in people aged 65 years and older, and (3) use of technology in the rehabilitation training in the older population. Systematic and narrative reviews were excluded. Based on consultation with the multidisciplinary research team, technological intervention studies were searched using the following search terms and the combination thereof: Parkinson, rehabilitation, technology, old*, elder*, effectiveness, randomized control trial. The detailed search strategy used in each database is reported in Multimedia Appendix 2. After the preliminary search, 190 papers resulted from PubMed, 71 from Embase, 87 from Scopus, 36 from CINAHL, and 1829 from Web of Sciences. Since the CINAHL database was consulted only after the first round of searches and after the duplicate analysis, a different filtering of the results was chosen. In fact, in order to narrow down the number of results, we looked for papers containing the term “randomized control trial” in the title. The findings were analyzed and screened by 4 experts of the team: a bioengineer, a clinical neurologist, a psychologist, and a physiotherapist. Rayyan software has been used in order to remove duplicates. Identified studies were independently reviewed for eligibility in a 2-step process; a first screening was performed by 4 independent authors (EM, GP, RB, and MB) based on the title and abstract. Then, full texts were retrieved for a second screening by the same 4 authors. At both stages, disagreements among reviewers were resolved by consensus and discussed with the other 2 authors (GA and VDD). After these steps, 25 papers resulted from selected databases. An additional researcher, with a background in biomedical engineering, confirmed the accuracy of the papers’ selection and screened for any possible omission. After the screening based on the inclusion or exclusion criteria, conducted on the full-text papers, 14 studies were selected. Figure 1 shows the flowchart search strategy applied. Data extraction, performed by 2 independent raters (EM and MB) included full reference details, sample size, study population details, including gender and mean age, type of technology used, outcomes, and results.

Quality appraisal of included studies was carried out by applying the PEDro scale suggested for evidence-based reviews [14]. The final score was settled when 3 authors reached agreement after repeated review and analysis.

### Data Synthesis

A descriptive synthesis of the data was performed. The data were grouped and analyzed based on the types of technological interventions used and their reported outcomes. The main areas of focus included cognitive and physical functioning, specifically gait and balance performance. The synthesis aimed to identify common findings and trends across the studies, highlighting the efficacy of technological interventions compared with traditional rehabilitation methods. No meta-analysis was conducted due to the heterogeneity of the study designs, interventions, and outcome measures.

## Results

### Study Quality Evaluation

A total of 14 papers were included [15-28]. Findings reported in this section are organized under macroconcept areas of interest.

Table 1 reports the quality evaluation of 14 population-based studies. In particular, the PEDro score ranged from 6 to a maximum of 8. The descriptive analysis of the technological intervention and devices used in the studies selected is shown in Multimedia Appendix 3.

**Table 1:** Scores of methodological quality assessment of the included studies.

PEDro	Eligibility	Randomized allocation	Concealed allocation	Baseline comparability	Blinded subject	Blinded therapists	Blinded raters	Key outcomes	Intention to treat	Comparison between groups	Precision and variability	Score
Del Din et al [15]	Y^a^	Y	Y	Y	N^b^	N	N	Y	Y	Y	Y	8/11
Jäggi et al [16]	Y	Y	Y	Y	N	N	N	Y	N	Y	N	6/11
Lau et al [17]	Y	Y	Y	Y	N	N	N	Y	N	Y	N	6/11
Kim et al [18]	Y	Y	Y	Y	N	N	N	Y	Y	Y	N	7/11
Maranesi et al [19]	Y	Y	Y	Y	N	N	Y	Y	N	Y	N	7/11
Gryfe et al [20]	Y	Y	Y	Y	N	N	N	Y	Y	Y	N	7/11
Feng et al [21]	Y	Y	Y	Y	N	N	Y	Y	N	Y	N	7/11
Cikajlo and Peterlin Potisk [22]	Y	Y	Y	Y	N	N	N	Y	N	Y	N	6/11
Capecci et al [23]	Y	Y	Y	Y	N	N	Y	Y	N	Y	N	7/11
Spina et al [24]	Y	Y	Y	Y	N	N	Y	Y	N	Y	N	7/11
Pazzaglia et al [25]	Y	Y	Y	Y	N	N	Y	Y	N	Y	N	7/11
Yuan et al [26]	Y	Y	Y	Y	N	N	Y	Y	N	Y	N	7/11
Calabrò et al [27]	Y	Y	Y	Y	N	N	Y	Y	Y	Y	N	8/11
Alagumoorthi et al [28]	Y	Y	Y	Y	N	N	Y	Y	Y	Y	N	8/11

^a^Y: yes.

^b^N: No.

### General Characteristics of the Study Population

All the studies were focused on older people with PD with a mean age of 69.8 (SD 14.4) years for the technological intervention group and mean age of 70.3 (SD 7.3) years in the control group. The number of participants involved in all the studies is 578, ranging from a minimum sample of 18 patients to a maximum of 128. There were 297 males and 281 females.

### Descriptive Analysis and Outcome Measures

Table 2 shows the characteristics of the included studies. All studies evaluated the impact of technological interventions on physical and cognitive domains. Regarding evaluated domains, cognitive functioning was assessed by 4 studies [16,17,20,22] and physical functioning was assessed by 13 studies [15,17-28], including gait assessment [15,17-20,23-25,27,28] in terms of gait speed or gait performance, falls and fear of falling evaluation [15,18,19,28], and balance estimation [16-18,20,21,24,28]. Examining the technology employed, there is a notable preference for using treadmills coupled with virtual reality and exergames [15,17,18,27]. In addition, numerous studies [16,19,23,24,28] incorporate sensitive pressure platforms, both static and dynamic, often in conjunction with nonimmersive virtual exercises. However, for the specific pathology under consideration, the use of exoskeletons [20] or gait robots [23] appears to be more restricted. It is worth noting that nearly all studies consistently incorporate virtual reality—whether immersive with visors or caves, or nonimmersive through exergames. This technology appears to have effects not only on the cognitive aspect but also on the physical domain. The analysis of the results clearly indicates that, in terms of gait and balance performance, the technological intervention outperforms the traditional approach, irrespective of the specific technology employed. However, from a cognitive perspective, the improvement is limited.

**Table 2:** Descriptive analysis of the included clinical studies.

	Population			Technology	Outcomes	Results
	Participants in EG^a^		Participants in CG^b^			
Del Din et al [15]	N=66 (older fallers with PD^c^); 81 male/47 female, aged 78.03 (6.21) years (older fallers with PD). HY^d^ stage: 2-3	N=62 (older fallers with PD)		Treadmill plus virtual reality	*Primary*: FRA^e^ index*.* * Secondary*: walking activity (total walking time per day, percentage of walking time per day, number of bouts, and steps per day)	*Between*: FRA was higher in PD compared with older fallers and MCI^f^ (*P*=.043). Walker activity was lower in MCI and patients with PD compared with older fallers (*P*<.012) * Within*: Walking activity did not change. FRA significantly decreased for all groups following both interventions (treadmill reduced FRA by 26%; treadmill + VR^g^ by 39%)
Jäggi et al [16]	N=19 (older people with PD); 12 male/7 female; aged 71.89 (9.09) years. HY stage: 1-4		N=21 (older people with PD); 15 male/6 female; aged 72.86 (10.14) years. HY stage: 1-4	Cognitive-motor training on the exergame device. Dividat Senso, a pressure-sensitive platform	*Primary*: feasibility (adherence rate; attrition rate; occurrence of adverse events; SUS^h^, NASA TLX^i^ score) * Secondary*: cognitive (Go/no go test; RTT^j^; D-KEFS^k^; TMT^l^); motor (gait speed; SPPB^m^; TUG^n^; 5×StS^o^)	*Primary*: Overall adherence rate was 96.5%. EG had an adherence rate of >70%. * Secondary*: no differences between EG and CG. Significant time-group interaction effects for 5×StS, SPPB, RTT, go/no go test, and D-KEFS
Lau et al [17]	N=9 (older people with PD); 6 male/3 female; aged 64 (9) years. HY stage: 1-3		N=9 (older people with PD); 6 male/3 female; aged 71 (5) years. HY stage: 1-3	Treadmill combined with a first-person immersive video game targeting visuospatial skills and working memory.	*Primary*: motor outcomes (6MWT^p^, TUG, TUG cognitive) * Secondary*: cognitive outcomes: (MoCA^q^, verbal fluency, SDMT^r^)	*Primary*: EG improves gait speed and walking distance during 6MWT, TUG cognitive (*P*=.05). EG improves TUG cognitive more that CG (between group: *P*=.04) * Secondary*: EG improves MoCA (*P*=.007) and SDMT (*P*=.01)
Kim et al [18]	N=22 (older people with PD); 6 male/16 female; aged 68.7 (6.9) years. HY stage: 2-3		N=22 (older people with PD); 7 male/15 female; aged 67.5 (9.3) years. HY stage: 2-3	Robot-assisted gait training (treadmill-based exoskeleton robot)	*Primary*: gait speed on the 10mWT^s^ * Secondary*: dual task interference on gait speed on the 10mWT, balance (TUG), disability score (BBS^t^), fear of falling (MDS-UPDRS^u^), freezing of gait (KFES-I^v^), brain functional connectivity changes (NFOGQ^w^)	*Primary*: no significant difference in gait speed on the 10mWT * Secondary*: EG shows significant improvements in BBS score and in MDS-UPDRS score. * Between*: EG shows significant difference in MDS-UPDRS
Maranesi et al [19]	N=16 (older people with PD); 6 male/10 female; aged 72.7 (6.3) years. HY stage: 2-3		N=14 (older people with PD); 5 male/9 female; aged 75.5 (5.4) years. HY stage: 2-3	Tymo system (wireless platform with nonimmersive virtual reality exergame)	*Primary*: POMA^x^ balance, POMA gait, and POMA total * Secondary*: gait speed, fear of falling (FES-I), autonomy in daily living activities (Barthel Index), and physical and psychological state of the patients (12-item Short Form Survey)	*Primary: Between*: POMA balance and POMA gait (EG vs CG) * Secondary*: *Between*: Barthel Index, 12-item Short Form Survey (EG vs CG)
Gryfe et al [20]	N=13 (older people with PD); 4 male/9 female; aged 67.6 (5.9) years. HY stage: 2-3		N=14 (older people with PD); 7 male/7 female; aged 70.7 (7.3) years. HY stage: 2-3	Gait robot (exoskeleton)	*Primary*: cognitive function (SCOPA-COG^y^) and mood * Secondary*: gait speed, 6MWT, freezing of gait, balance, and quality of life	*Primary*: significant improvement in SCOPA-COG in EG than in CG * Secondary*: significant improvement in 6MWT in EG than in CG
Feng et al [21]	N=14 (older people with PD); 7 male/7 female; aged 67.5 (4.8) years. HY stage: 2-4		N=14 (older people with PD); 8 male/6 female; aged 66.9 (4.6) years. HY stage: 2-4	Virtual reality technology	Motor ability (BBS, TUG, UPDRS-III, and FGA^z^)	*Within*: BBS, TUG, and FGA improve significantly in EG and CG (*P*<.05). *Between groups*: BBS, TUG, UPDRS-III, and FGA in EG are better than in EG (*P*<.05).
Cikajlo et al [22]	N=10 (older people with PD), 4 male/6 female, aged 71.3 (8.4) years		N=10 (older people with PD), 5 male/5 female, aged 67.6 (7.6) years	Immersive VR using 3D Oculus Rift CV1	*Primary*: effectiveness of treatment (BBT, UPDRS) * Secondary*: motivation effect (Motivation Inventory)	*Primary*: time of manipulation (group × time, *P*=.009), number of successfully placed cubes (group × time, *P*=.028), average tremor (group × time, *P*=.002), and UPDRS for upper limb (U3=0.35). The LCD and 3D groups substantially improved their BBT score with training (U3=0.7, U3=0.6, respectively).
Capecci et al [23]	N=48 (older people with PD), 19 male/29 female, aged 68.1 (9.8) years. HY stage: ≥2		N=48 (older people with PD), 24 male/24 female, aged 67.0 (7.6) years. HY stage: ≥2	GE-O system	*Primary*: motor function (6MWT, TUG, 10MWT, minimal clinically important difference) * Secondary*: Freezing of Gait Questionnaire, UPDRS	Both groups showed significant improvement in all outcomes. As compared with baseline, with robot-assisted gait training and treadmill training, endurance and gait capacity were enhanced by 18% and 12%, respectively, and motor symptoms and quality of life were improved by 17% and 15%, respectively. The maximum advantage was observed with the Freezing of Gait Questionnaire score, which decreased by 20% after either treatment.
Spina et al [24]	N=11 (older people with PD), 5 male/6 female, aged 68 (6.9) years. HY stage: 1-2		N=11 (older people with PD), 4 male/7 female, aged 67.27 (4.85) years. HY stage: 1-2	Hunova (robotic platform)	*Primary*: Quantified balance impairments (mini-BESTest, BBS) * Secondary*: Motor ability (10MWT, 5×StS, PDQ-39^aa^, TUG)	*Between*: no significant differences * Within*: primary outcomes improved in EG and CG.
Pazzaglia et al [25]	N=25 (older people with PD), 18 male/7 female, aged 72 (7) years		N=26 (older people with PD), 17 male/9 female, aged 70 (10) years	Nirvana (VR system)	*Primary*: changes in functional standing balance (BBS) * Secondary*: ability to adapt gait to complex walking tasks (DGI^ab^); physical function of the upper limb (DASH^ac^); and physical and emotional scores (SF-36^ad^)	*Between*: in EG increases BBS score (*P*=.003), DGI score (*P*=.003), and SF-36 mental composite score (*P*=.037), and a decrease in DASH scale score (*P*=.009). In CG DASH scale score decreases (*P*=.007).
Yuan et al [26]	N=12 (older people with PD), 2 male/10 female, aged 67.8 (5.5) years. HY stage: 1-3		N=12 (older people with PD), 9 male/3 female, aged 66.5 (8.8) years. HY stage: 1-3	Video game–based treatment	*Primary*: Quantified balance impairments (BBS score) * Secondary*: Quality of life and motor/balance ability (SF-36, MFES^ae^, MDRT^af^, and Maximum Step Length test)	*Between*: changes in MFES and MDRT to the right and left sides were significantly different in the first 6-week period. Changes in BBS, MFES, and MDRT to the right and left sides were significantly different in the second 6-week period.
Calabrò et al [27]	N=25 (older people with PD), 11 male/9 female, aged 70 (8) years. HY stage: 2-3		N=25 (older people with PD), 9 male/3 female, aged 73 (8) years. HY stage: 2-3	Treadmill plus music	*Primary*: gait performance (FGA) * Secondary*: brain oscillation changes related to gait cycle; gait and motor performance: (UPDRS, BBS, FES, 10MWT, TUG, and GQI^ag^)	*Between*: improvement in FGA (*P*<.001), FES (*P*<.001), UPDRS (*P*=.001), and overall GQI (*P*<.001) in EG
Alagumoorthi et al [28]	N=96 (with PD), 51 male/45 female, aged 69.7 (10) years. HY stage 2-3-4		N=96 (with PD), 63 male/33 female, aged 68.5 (9.8) years. HY stage: 2-3-4	Nintendo Wii Console	*Primary*: number of fallers * Seconda*ry: fall rate; risk of falling and quality of life (BBS, TUG, and PDQ-39)	End of treatment: *Between*: number of fallers (*P*=.77), BBS (*P*=.658), TUG (*P*=.967), and PDQ-39 (*P*=.402). Follow-up 36th week: *Between*: number of fallers (*P*=.039), BBS (*P*=.867), TUG (*P*=.959), and PDQ-39 (*P*=.405).

^a^EG: experimental group.

^b^CG: control group.

^c^PD: Parkinson disease.

^d^HY: Hoehn & Yahr.

^e^FRA: Falls Rate and Activity Exposure Index.

^f^MCI: mild cognitive impairment.

^g^VR: virtual reality.

^h^SUS: System Usability Scale.

^i^NASA TLX: NASA Task Load Index.

^j^RTT: reaction time test.

^k^D-KEFS: Delis-Kaplan Executive Function System; color-word interference test.

^l^TMT: Trail Making Test A and B.

^m^SPPB: Short Physical Performance Battery.

^n^TUG: time up and go.

^o^5×StS: 5 times sit to stand.

^p^6MWT: 6-minute walking test.

^q^MoCA: Montreal Cognitive Assessment.

^r^SDMT: Symbol Digit Modality Test.

^s^10mWT: 10-minute walking test.

^t^BBS: Berg Balance Scale.

^u^MDS-UPDRS: Movement Disorder Society-sponsored version of the Unified Parkinson’s Disease Rating Scale.

^v^KFES-I: Korean version of Falls Efficacy Scale-International.

^w^NFOGQ: New Freezing of Gait Questionnaire.

^x^POMA: Tinetti Performance Oriented Mobility Assessment.

^y^SCOPA-COG: Scales for Outcomes in Parkinson’s Disease-Cognition.

^z^FGA: Functional Gait Assessment.

^aa^PDQ-39: 39-item Parkinson’s Disease Questionnaire.

^ab^DGI: Dynamic Gait Index.

^ac^DASH: Disabilities of the Arm, Shoulder and Hand.

^ad^SF-36: 36-item Short Form.

^ae^MFES: Modified Falls Efficacy Scale.

^af^MDRT: Multi-Directional Reach Test.

^ag^GQI: Gait Quality Index.

## Discussion

### Principal Findings

This systematic review seeks to shed light on the evolving landscape of technology-assisted rehabilitation for older people with PD. Delving into the available evidence, we assess how technology can serve as a valuable complement to conventional therapy, offering new ways to optimize care and improve outcomes in this growing patient demographic. In particular, the included studies evaluated the impact of technological interventions on physical and cognitive domains. Cognitive functioning was assessed in 4 studies, while physical functioning, including gait, falls, and balance, was assessed in 13 studies. Technologies used included treadmills with virtual reality, exergames, and sensitive pressure platforms. Exoskeletons and gait robots were less commonly used. Most studies incorporated virtual reality, either immersive or nonimmersive. The results indicate that technological interventions significantly improve gait and balance performance compared with traditional methods, though cognitive improvements were limited.

PD poses a serious burden on patients, carers, families, health care providers, and health authorities globally. Within the relevant unmet need in the management of PD [10], identifying preventive strategies, particularly tertiary, to mitigate severe progression and ensure an adequate quality of life for older patients is one of the most felt needs, with the final aim of slowing disease progression, enhancing the long-term quality of life, and reducing the costs associated with PD, as the majority of expenses occur in advanced stages.

From our analysis, it seems that including technology into a physical intervention can constitute a driver for ensuring the adherence and compliance to rehabilitation trainings. In particular, our analysis reveals an overall improvement in the physical status and outcomes related to disease severity, even if a not clear improvement in cognitive status and quality of life was observed. This result may suggest the need of conducting longer trials to collect evidence on the impact of rehabilitative interventions with technology also in the long term. Moreover, even if the adherence to rehabilitation training is high in hospital facilities, this should be complemented and sustained with training at home to stimulate compliance to physical exercise, considering that patients with PD should remain active as long as possible to counteract the disease progression. Additionally, digital solutions can be a viable option to support the older patients at home by coaching them in an interactive and engaging way, as already demonstrated by numerous evidence in the field [29].

In fact, although in a different context, our analysis has shown that the most engaging interventions involved virtual reality technology, which appears to be the most promising tool for positively impacting cognitive domains due to its highly interactive features. This is in line with recent findings that highlight that virtual reality technology has a particularly promising tool for investigating and rehabilitating gait and balance impairments in people with PD by enabling users to engage in an enriched and highly personalized complex environment [30].

The inclusion of technology and virtual reality into rehabilitative training imposes to stress the attention to assessing the usability and accessibility of such technologies by including the patients-in-the-loop since the design of the solutions in order to be suitable and to not compromise the appropriate use. At this purpose, even if there is a plethora of studies and disciplines that underline the needs of involving older adults and vulnerable user groups in the design process, also with a specific focus on age-related conditions such as dementia [29], there has been limited exploration of how individuals with PD can actively participate in the design process [31], despite the fact that the PD population represents an intriguing user group for design investigation due to the intricate and individual nature of the condition that includes physical and cognitive symptoms, with fluctuations in severity, while dealing with emotional challenges related to social stigma and embarrassment associated with their condition [32].

However, this systematic review has its limitations. The low number of studies and their heterogeneity do not favor valid and generalizable conclusions, but this very limitation shows that, despite the good promises, there is still a need to experiment with technological interventions and, in particular, with virtual reality, in the rehabilitation of people with PD. Moreover, all the studies reported show more or less significantly positive results: this aspect is typical of scientific publications, in which there is a tendency to publish this kind of study to the detriment of those with negative results. For the purposes of research into the implementation of new technologies for the treatment of patients with PD, it is very important to know and take into account even those studies that did not produce the desired results, so that lessons should not be learned again.

The integration of technology into PD rehabilitation holds the promise of enhancing the efficacy and accessibility of interventions, addressing the unique challenges faced by older individuals, and facilitating personalized, patient-centered care. Future challenges of sociotechnical interventions should be focused on unmet needs of the older population with PD by combining a multidisciplinary, multicomponent, and personalized approach and addressing challenges related to health literacy, availability of physical and psychological services, and the stigma associated with PD [10].

### Conclusions

In summary, this systematic review seeks to shed light on the evolving landscape of technology-assisted rehabilitation for older individuals with PD. The integration of technology into PD rehabilitation holds the promise of enhancing the efficacy and accessibility of interventions, addressing the unique challenges faced by older individuals, and facilitating personalized patient-centered care.

## Appendix

Multimedia Appendix 1 PRISMA (Preferred Reporting Items for Systematic Reviews and Meta-Analyses).

Multimedia Appendix 2 Search strategy used in each database.

Multimedia Appendix 3 Descriptive analysis of the technology used in the interventions analyzed.

## Abbreviations

PD: Parkinson disease
PRISMA: Preferred Reporting Items for Systematic Reviews and Meta-Analyses
