# Supplementary material for: Effectiveness of Technological Interventions for Older Adults With Parkinson Disease: Systematic Review
Source: JMIR Serious Games. 2024 Sep 9;12:e53431. doi: 10.2196/53431 (PMC11430395; doi:10.2196/53431)
Supplement: Multimedia Appendix 2 [file games_v12i1e53431_app2.docx]

**PUBMED**

((parkinson[MeSH Terms] OR parkinson[Title/Abstract] AND rehabilitation[Title/Abstract] OR "physical rehabilitation"[Title/Abstract] AND (“technology"[Title/Abstract] OR "technology"[MeSH Terms] OR "effectiveness"[MeSH Terms])) AND ("randomized controlled trial"[Publication Type] OR "randomized controlled trials as Topic"[MeSH Terms] OR RCT[Title/Abstract] OR RCTs[Title/Abstract] OR "randomized control trial*"[Title/Abstract] OR "randomised control trial*"[Title/Abstract]) AND (old*[Title/Abstract] OR elder*[Title/Abstract])

**EMBASE**

((('parkinson'/exp OR ‘parkinson’:ti,ab) AND (‘rehabilitation'/exp OR 'rehabilitation’:ti,ab) AND (‘technology'/exp OR 'technology’:ti,ab) AND (‘old*'/exp OR 'old*’:ti,ab OR ‘elder*'/exp OR 'elder*’:ti,ab) AND ('randomized controlled trial'/exp OR RCT:ti,ab OR RCTs:ti,ab OR ‘randomized control trial*’:ti,ab OR ‘randomised control trial*’:ti,ab**)**

**WEB OF SCIENCE**

(((KP=parkinson OR KP=rehabilitation OR (TI=rehabilitation OR AB=rehabilitation) OR (TI=technology OR AB=technology)) AND ((TI=effectiveness OR AB=effectiveness) OR KP="old*" OR KP="elder*")) OR KP=”eHealth literacy” OR KP=”digital health literacy”) AND (KP="randomized controlled trial*" OR KP="non-randomized controlled trial*" OR KP="randomised controlled trial*" OR (TI=RCT OR AB=RCT) OR (TI=RCTs OR AB=RCTs) OR (TI="randomized control trial*" OR AB="randomized control trial*") OR (TI="randomised control trial*" OR AB="randomised control trial*")

**SCOPUS**

((INDEXTERMS(parkinson) OR TITLE-ABS(parkinson) OR TITLE-ABS(PD) OR TITLE-ABS(technology)) AND (TITLE-ABS("rehabilitation") OR INDEXTERMS(parkinson) OR INDEXTERMS(technology) OR INDEXTERMS(effectiveness) OR INDEXTERMS(old*) OR INDEXTERMS(elder*))) AND (INDEXTERMS("randomized controlled trials") OR TITLE-ABS(RCT) OR TITLE-ABS(RCTs) OR TITLE-ABS("randomized control trial*") OR TITLE-ABS("randomised control trial*")

**CINAHL**

((MH "Parkinson's Disease" OR TI "Parkinson*" OR AB "Parkinson*") AND (TI "Rehabilitation" OR AB "Rehabilitation" OR TI "Physical Rehabilitation" OR AB "Physical Rehabilitation") AND (TI "Technology" OR AB "Technology" OR MH "Technology" OR TI "Technological" OR AB "Technological") AND (TI "Effectiveness" OR AB "Effectiveness" OR MH "Effectiveness" OR TI "Efficacy" OR AB "Efficacy" OR TI "Impact" OR AB "Impact") AND (TI "Randomized Controlled Trial" OR AB "Randomized Controlled Trial" OR MH "Randomized Controlled Trials as Topic" OR TI "RCT" OR AB "RCT" OR TI "RCTs" OR AB "RCTs" OR TI "Randomized Control Trial*" OR AB "Randomized Control Trial*" OR TI "Randomised Control Trial*" OR AB "Randomised Control Trial*") AND (TI "Old*" OR AB "Old*" OR TI "Elder*" OR AB "Elder*"))
