# Supplementary material for: Effectiveness of Technological Interventions for Older Adults With Parkinson Disease: Systematic Review
Source: JMIR Serious Games. 2024 Sep 9;12:e53431. doi: 10.2196/53431 (PMC11430395; doi:10.2196/53431)
Supplement: Multimedia Appendix 3 [file games_v12i1e53431_app3.docx]

**Table S1.** Descriptive analysis of the technology used in the interventions analyzed.

|  | **Technology** | **Intervention** |
| --- | --- | --- |
| Del Din et al., [13] | Treadmill plus virtual reality. | 18 sessions (3 times per week for 6 weeks). 40-minutes walking on treadmill with or without VR. Falls rate was recorded at baseline and during the 6 months after the end of the training. Participants were asked to wear a tri-axial accelerometer-based wearable device for 7 days 4 times (pre-intervention, after 1 week, after 4 weeks, after 6 months) to analyse their gait. |
| Jäggi et al., [14] | Dividat Senso, a pressure-sensitive platform with 20 strain-gauges, 5 vibration motors and an LED control; it is wired with a screen where the exergame is projected; the user is required to move steps in four directions or shift the body weight to execute the actions. | Participants played 5 to 7 games (about 15 minutes in total) for 5 times a week; the difficulty of the game was adapted automatically and real-time by the embedded DividatPlay software, but the investigator could slightly adjust it. |
| Lau et al., [15] | Split-belt treadmill combined with a large screen where the walking scenario is projected; three buttons in front of the user allow him/her to control forward/reverse, right, and left turns; clinician set the inclination of the treadmill and the speed of each leg, according to user’s need. | 12 sessions (3 times per week for 4 weeks). Walking on the treadmill for 30 minutes with a break in between. |
| Kim et al., [16] | Walkbot-S, a robot-assisted gait training (treadmill-based exoskeleton robot), capable of delivering continuous passive motion to the legs. During the RAGT process, participants received an auditory cue during the toe-off phase to enhance their gait rhythm. Simultaneously, a front-facing screen provided visual feedback in the form of torque graphs for each leg. Positive feedback was displayed when the torque reached the predetermined target range of the robot's joint. | 12 sessions (3 times per week for 4 weeks; each session was 45 min, including 15 min of warm-up and cool-down. The gait velocity was increased session by session according to the user’s capabilities. |
| Maranesi et al. [17] | Tymo system is a platform combined with a non-immersive virtual reality exergames; the user has to move to reach the targets on the game. The clinician decides if the user has to move in one or two dimensions. Games are from Verena Schweizer’s neurotraining. | 10 sessions (2 sessions per week for 5 weeks); each session lasted 50 minutes: 30 minutes of traditional therapy and 20 of technological intervention. |
| Gryfe et al. [18] | Keeogo Rehab™ bilateral exoskeleton | 16 sessions (2 sessions per week for 8 weeks). Participants wore the Exo for the whole session, that was composed of: walking warm up, repeated sit-to-stands, forward and reverse lunges, aerobic walking, stairs, step-up/down, backward and side stepping, heel rises, stepping, rotating, reaching, obstacle course. |
| Feng et al., [19] | Virtual reality technology: warm up, cool down, and three games in between: hands and feet touch the ball, hard boating, and take the maze. | 60 sessions (5 sessions per week for 12 weeks). Sessions were performed 2 hours after medication, for 45 minutes (5 minutes warm up, 3 x 10-minute games, 10 minutes for cooling down). Medication: 16 patients took dopamine agonists and 12 patients took L-dopa-Bensom and/or levodopa-carbidopa. |
| Cikajlo et al., [20] | Immersive VR using 3D Oculus Rift CV1, with the developed 10Cubes exergame: colored cubes to be picked and placed by virtual hand with fingers, enabling also a pinch grip. | 10 sessions (30 minutes long) in 3 weeks, where using the most affected hand to execute the action. 1-2 minutes break between each game session. |
| Capecci et al., [21] | GE-O system, an end-effector robotic device that helps in walking by partially supporting the body weight and guiding the movement of the lower limbs. | 20 sessions (5 sessions per week for 4 weeks): robot-assisted walking at variable speeds for 45 min; the speed was gradually increased while the body weight support was gradually decreased. |
| Spina et al., [22] | Hunova composed of two electromechanical robotic platforms, with 2 degree of freedom: under the feet and under the seat, to allow both standing and sitting exercises. The platforms can move or not, allowing static and dynamic modes. Thanks to IMU, the device can track the body segments. | 20 sessions (5 sessions per week for 4 weeks). Each session consisted of 10 standing position exercises (bipodal mode exercises) followed by 10 sitting position exercises in both static and dynamic modes. The subject had to maintain the centre of pressure in a defined area of confidence, or had to stabilize the upper trunk with both static and dynamic seat/base or during random perturbations offered by the device. The difficulty increased session by session. |
| Pazzaglia et al., [23] | Nirvana, a markerless system for motion analysis, utilizing optoelectronic infrared devices, enabling patients to engage in exercises within virtual environments while experiencing complete audio-visual sensory immersion. | 18 sessions (3 sessions per week for 6 weeks). Each session lasted 40 minutes: warm up, sitting and standing exercises for motor coordination of limbs, balance, and walking training, and cool down. |
| Yuan et al., [24] | XaviX entertainment system: video game-based treatment, offers aural and visual feedback in both multi-directional step task and target-directed stepping task. The patient moves on a platform to accomplish the tasks showed on a connected screen. | 18 sessions (3 sessions per week for 6 weeks). Each session lasted 30 minutes (15 minutes each task). Difficulty adjusted by the clinician. |
| Calabrò et al., [25] | A walking treadmill equipped with rhythmic auditory stimulation (RAS) | 40 sessions (5 times per week for 8 weeks): 45 min of conventional overground gait training, 45 min of activities in daily living training and reaching activities in occupational therapy, 45 min of biomechanical training in both the upper and lower limbs, 30 min of speech therapy, and 30 min of rest distributed between the sessions (for a total of 195 min). Then, the individuals were provided with further 30 min of RAS treadmill training. |
| Alagumoorthi et al. [26] | Nintendo Wii Console | 36 sessions (3 times per week for 12 weeks). Traditional therapy + 30 – 40 min of Wii-sport based strategy training. Participants played 2 – 3 minutes per game (boxing, base -ball pitching, bowling, table tennis, sword play, golf, base-ball bating, frisbee, bowling, and tennis) in standing position. |
